# Kentucky State Dental Association

**Published:** 1882-09

**Authors:** Chas. E. Dunn, J. Hooper

**Affiliations:** President


### KENTUCKY STATE DENTAL ASSOCIATION.

Twelfth annual meeting of the Kentucky State Dental Association, held in the Academy Room of the Polytechnic Society, Louisville, Ky., June 6, 1882.

The Association was called to order by the president, Dr. W. W. Justice, at 2:¾ o'clock P.M., and the meeting opened with prayer by Dr. W. H. Morgan, of Nashville, Tenn. The roll was called, and the following were present: President Dr. W. W. Justice, Vice-President Dr. J. Hooper, Secretary Dr. C. E. Dunn, Treasurer Dr. G. F. Canine, Dr. W. N. Alsop, M. L. S. Buckner, C. G. Edwards, W. H. Goddard, F. L. Klengman, F. Peabody, A. O. Rawls, B. G. Reese, and Dr. W. H. Morgan (honorary member) of Nashville.

The president appointed Drs. Peabody and Edwards as members *pro tem*, of the Board of Censors, in place of Drs. Kidd and R. C. Morgan.

The minutes of the last annual meeting were read and approved.

On the suggestion of Dr. Edwards the president deferred the reading of his address until to-morrow (Wednesday) morning at 10 o'clock.

The petition of W. S. Smith, of Newcastle, for membership was received and referred to the Board of Censors.

Dr. Goddard announced the death of Drs. M. S. Dean, of Chicago, Ill., and James Taylor, of Cincinnati, O., and moved the appointment of a committee on demises, and the president appointed Drs. W. H. Morgan, F. Peabody and A. O. Rawls. Dr. Morgan spoke in feeling terms of the great and useful work these gentlemen had done for our profession.

The amendment to the Constitution, offered at last annual meeting by Dr. A. W. Smith, to wit: Amend Article 2, Sec. 4 by striking out the words "their diplomas and" was on motion adopted. The Section now reads: "All members must be elected by ballot, requiring an affirmative vote of two-thirds of all present to entitle them to admission, and after signing the Constitution, and paying the fees, shall be entitled to all the privileges of the Association."

The Board of Censors made a report on the petition of W. S. Smith, recommending him for membership. On motion, the report was received, and the ballot taken. Dr. Smith was unanimously elected as a member of the Association.

On motion, adjourned to meet to-morrow at 9½ A.M.

SECOND DAY—Wednesday, June 7, 1882.

The meeting was called to order by the president, at 9¾ o'clock.

The following members and visitors were present, in addition to those of yesterday: Drs. H. A. Smith, James I. Taylor, and A. Cutter, of Cincinnati, O.; Dr. W. W. Evans, Washington City; Drs. W. F. Morrill, F. C. Greene, of Indiana; W. M. Garnett, L. F. Huffman, J. B. McGee, W. G. Redman, of Kentucky.

The president, Dr. Justice, read his address, which, on motion, was referred to a committee of three—Drs. Edwards, R. C. Morgan, and W. H. Morgan, for division of subject requiring action of the Association.

The request for membership of Drs. L. F. Huffman, W. M. Garnett, J. B. McGee, and C. E. Canine were received and referred to the Board of Censors.

The report of the Board of Censors, recommending the above named gentlemen, was, on motion, received, and the ballot was taken separately, and they receiving the unanimous vote, and were severally declared elected.

On motion of Dr. Dunn, Dr. Evans, of Washington, was invited to give a clinic on his method of working celluloid, to-morrow (Thursday), morning at 10 o'clock.

On motion, adjourned.

THIRD DAY.—Thursday, June 8, 1882.

The Association was called to order at 10 o'clock A.M.

This being the hour appointed for clinic, Dr. Evans gave a demonstration on celluloid with the "new mode heater," with which all were very much gratified.

Present, in addition to those reported previously, Prof. J. Taft, Drs. Alphonso Taft, of Cincinnati, O.; A. W. Smith, of Richmond; Eggers, of Louisville.

The committee to whom was referred the President's address, made the following report, which was, on motion, accepted.

Your committee on the President's address would respectfully report—

That the address is full of interest to the profession, and is received with pleasure by the Association, and that we can not too highly commend the subject of dental education, especially to the younger members and the student.

The reference to ancient dentistry is of especial interest, and if practical we would suggest the appointment of a committee to investigate as fully as possible everything relating to the early efforts in this direction, the labors of which have resulted in a scientific profession.

C. G. Edwards,<br>R. C. Morgan<br>W. H. Morgan, } Committee.

The following resolution, offered by the Executive Committee, was adopted unanimously:

*Resolved*—That the thanks of the Association be, and are, hereby tendered to the President and Executive Committee of the Polytechnic Society of Kentucky, for the free use of the Academy Room, belonging to said institution, for our present annual meeting.

On motion, proceeded to election of officers, which resulted as follows:

President—Dr. J. Hooper, of Owenton.

Vice-President—Dr. R. C. Morgan, of Stanford.

Treasurer—Dr. J. F. Canine, of Louisville.

Secretary—Dr. Chas. E. Dunn, of Louisville.

Member Board of Censors—Dr. A. O. Rawls, of Lexington.

Member Board of Censors—Dr. M. L. S. Buckner, of Shelbyville.

State Board of Examiners—Dr. A. W. Smith, Richmond.

Member of Executive Committee—Dr. A. W. Smith, of Richmond.

Lexington was chosen as the place for holding the next annual meeting.

Adjourned to meet to-morrow (Friday), morning at 9 o'clock.

The afternoon was taken up by visiting the Lithgow Manufacturing Company, the Pictet Ice Company, and points of interest in the city.

FOURTH DAY—Friday, June 9, 1882.

The Association was called to order by the President at 9½ o'clock.

The Treasurer made his annual report, which was, on motion, received and referred to the Executive Committee.

The Executive Committee reported they had examined the Treasurer's report and found the same correct. On motion, the report was received.

Dr. W. H. Morgan, Chairman of Committee on Demises, having been compelled to leave, Dr. J. Taft was added to the committee, and they were given leave to hand their report to the Secretary after close of the meeting, and have it published in the dental journals.

The following resolutions offered by Dr. Goddard, were, on motion, unanimously adopted:

*Resolved*—That the thanks of this Association be, and are, hereby tendered the Lithgow Manufacturing Company, the Pictet Ice Company, Dr. W. W. Evans, of Washington, D.C., the Louisville *Courier-Journal*, *Commercial*, *Evening Post*, Prof. Thos. W. Tobin, and all others who have in any way contributed to the interest and advancement of the Association.

*Resolved*—That the thanks of the Association be, and are, hereby tendered the retiring officers for the faithful manner in which they have discharged the duties of their office.

On motion of Dr. Dunn, (he having voted in the affirmative), the motion adopted selecting Lexington as the place for the next annual meeting, was reconsidered.

On motion of Dr. Hooper, Louisville was selected as the place for the next annual meeting.

The following amendment to the Constitution was submitted and ordered laid over until the next annual meeting.

Amend Section 2, Article 1, by inserting the words—"In the city of Louisville," and strike out the words "at such place as may be designated by the Association at the time of election, the preceding year. Signed,

J. Hooker,

A. Wilkes Smith,

W. S. Smith.

Wm. Garnett,

W. W. Justice.

Dr. Goddard stated that according to the laws of the American Dental Association the delegates to that Association must be graduates of a dental college to be admitted, and he understood two of the delegates elected yesterday were not graduates, and thought the Association had better elect others in their place. Dr. C. E. Canine and W. M. Garnett were put in nomination and duly elected in place of Drs. Justice and Huffman.

Drs. Alphonso Taft, of Cincinnati, O.; F. C. Greene of New Albany, Ind.; J. W. Baxter, Jr., of Vevay, Ind., and Prof. T. W. Tobin, of Louisvllle, Ky., were proposed for honorary membership, and referred to the Board of Censors.

The Board of Censors reported, recommending the above named gentlemen. On motion, the report was received, and the ballot taken, they receiving the unanimous vote were severally declared elected honorary members.

Letter of B. H. Young, President of Polytechnic Society was received and placed on file.

On motion, the Association adjourned to meet in the city of Louisville on the first Tuesday of June, 1883.

Chas. E. Dunn, Secretary. J. Hooper, President.

